# Supplementary material for: Self-bridging metamaterials surpassing the theoretical limit of Poisson’s ratios
Source: Nat Commun. 2023 Jul 7;14:4041. doi: 10.1038/s41467-023-39792-9 (PMC10328922; doi:10.1038/s41467-023-39792-9)
Supplement: Supplementary file 1 — Supplementary Information [file 41467_2023_39792_MOESM1_ESM.pdf]

Supplementary Information

Self-bridging metamaterials surpassing the theoretical limit of Poisson's ratios

Jinhao Zhang[1], Mi Xiao[1]*, Liang Gao[1]*, Andrea Alù[2], Fengwen Wang[3]

[1]State Key Laboratory of Digital Manufacturing Equipment and Technology, Huazhong University of Science and Technology, 430074 Wuhan, China

[2]Photonics Initiative, Advanced Science Research Center, City University of New York, New York, New York 10031, USA

[3]Department of Civil and Mechanical Engineering, Technical University of Denmark, Koppels Allé, Building 404, 2800 Kongens Lyngby, Denmark

*Corresponding author. Email: xiaomi@hust.edu.cn, gaoliang@mail.hust.edu.cn

Supplementary Note 1

Non-reciprocal Poisson's ratios

The compliance matrix **C** in the orthotropic linear elastic regime is

$$\mathbf{C}=\begin{pmatrix} 1/E_1 & -\nu_{12}/E_2 & 0 \\ -\nu_{21}/E_1 & 1/E_2 & 0 \\ 0 & 0 & 1/G \end{pmatrix} \tag{1}$$

where E_1 and E_2 are the elastic moduli in the x_1 and x_2 directions, respectively. G is the shear modulus. The symmetry of the compliance matrix gives

$$E_1\nu_{12}=E_2\nu_{21} \tag{2}$$

The elastic modulus (E_1 or E_2) can be calculated by using the stress (F_1/A or F_2/A) and strain (u_1/a or u_2/a), where F_1 (F_2) is the reaction force of the unit cell with periodic boundary conditions under the strain u_1/a (u_2/a). $A=at$, where t is the thickness of the unit cell. Then, $E_1\nu_{12}=E_2\nu_{21}$ can be rewritten as

$$\frac{F_1 a}{u_1 A}\nu_{12}=\frac{F_2 a}{u_2 A}\nu_{21} \tag{3}$$

$$F_1 v_{12} u_2 = F_2 v_{21} u_1 \tag{4}$$

Based on $v_{12} = -u_1 / u_2$ and $v_{21} = -u_2 / u_1$, the input displacement u_2 (u_1) in the x_2 (x_1) direction outputs the displacement $u_{2\to1}$ ($u_{1\to2}$) in the x_1 (x_2) direction. The relations between Poisson's ratios and these displacements are

$$-v_{12} u_2 = u_{2\to1} \tag{5}$$

$$-v_{21} u_1 = u_{1\to2} \tag{6}$$

Then, Supplementary Equation (4) can be expressed as a static reciprocal formulation:

$$F_1 u_{2\to1} = F_2 u_{1\to2} \tag{7}$$

For a general continuum with positive elastic moduli E_1 and E_2, both v_{12} and v_{21} are positive, zero, or negative simultaneously; then, $v_{ij} v_{ji} \geq 0$. Based on Supplementary Equations (2) and (7), surpassing this limit to make $v_{21} > 0$ and $v_{12} < 0$ can offer an unusual deformation pattern, i.e., non-reciprocal transmission of displacement fields (Fig. 1c and f), where $E_2 v_{21} > 0 > E_1 v_{12}$ and $F_1 u_{2\to1} > 0 > F_2 u_{1\to2}$. Then, in this work, $v_{ij}v_{ji} < 0$ are called non-reciprocal Poisson's ratios.

Supplementary Note 2

Ultra-large Poisson's ratios

Mathematically, the compliance matrix has to be positive-definite to ensure a positive strain energy density. Then, the Poisson's ratios in the linear orthotropic constitutive law are thermodynamically constrained by $v_{ij} v_{ji} < 1$. Hence, Poisson's ratios larger than 1 in both orthogonal directions and the deformation pattern of orthogonally bidirectional displacement amplification (Fig. 1d and f) are inaccessible in the linear elastic regime. Then, in this work, $v_{ij} v_{ji} > 1$ are called ultra-large Poisson's ratios.

Supplementary Note 3

Step-like Poisson's ratios

The internal connectivities of a continuum are unchanged under a small strain in the linear elastic regime, and the constitutive relation is mathematically invariant. Consequently, the Poisson's ratio in the linear elastic regime does not change either under tensile or compressive strain in a particular direction, which is demonstrated by a line with a slope of 1 in Fig. 1f. Hence, ordinary materials in the linear elastic regime cannot achieve step-like Poisson's ratios that are negative and positive under longitudinal tension and compression, respectively, and cannot exhibit the deformation pattern of transverse expansion under both load cases (Fig. 1e). Then, in this work, step-like Poisson's ratios are defined as compressive $v_{ij} > 0$ and tensile $v_{ij} < 0$.

Supplementary Note 4

Effective material parameters

The representative volume element method is used to calculate the effective elastic matrix $\mathbf{D}^*$.

$$\mathbf{D}^* = \begin{pmatrix} D_{11}^* & D_{12}^* & 0 \\ D_{21}^* & D_{22}^* & 0 \\ 0 & 0 & G^* \end{pmatrix} \tag{8}$$

With an average strain ($\overline{\varepsilon_1}$, $\overline{\varepsilon}_2$, or $\overline{\varepsilon}_{12}$) applied to the unit cell, the effective elastic matrix can be calculated using the corresponding average stress ($\overline{\sigma}_1$, $\overline{\sigma}_2$, or $\overline{\sigma}_{12}$), which is given as

$$\frac{S}{a^2}\overline{\sigma}_1 = D_{11}^*\overline{\varepsilon}_1 \tag{9}$$

$$\frac{S}{a^2}\overline{\sigma}_1 = D_{12}^*\overline{\varepsilon}_2 \tag{10}$$

$$\frac{S}{a^2}\overline{\sigma}_2 = D_{22}^*\overline{\varepsilon}_2 \tag{11}$$

$$\frac{S}{a^2}\bar{\sigma}_{12} = 2G^*\bar{\varepsilon}_{12} \tag{12}$$

where S is the area of the solid in a unit cell. Based on $\mathbf{C}^* = \mathbf{D}^{*-1}$, the effective compliance matrixes $\mathbf{C}^*$ of the equivalent models in Supplementary Fig. 4 are obtained as follows:

$$\begin{pmatrix} 2.55\text{E}-6 & -3.29\text{E}-6 & 0 \\ -3.29\text{E}-6 & 1.59\text{E}-5 & 0 \\ 0 & 0 & 9.26\text{E}-6 \end{pmatrix} \tag{13}$$

$$\begin{pmatrix} 1.34\text{E}-6 & 5.94\text{E}-7 & 0 \\ 5.94\text{E}-7 & 1.34\text{E}-6 & 0 \\ 0 & 0 & 6.76\text{E}-6 \end{pmatrix} \tag{14}$$

$$\begin{pmatrix} 3.77\text{E}-7 & -7.26\text{E}-7 & 0 \\ -7.26\text{E}-7 & 9.66\text{E}-6 & 0 \\ 0 & 0 & 1.11\text{E}-5 \end{pmatrix} \tag{15}$$

$$\begin{pmatrix} 9.66\text{E}-6 & -7.26\text{E}-7 & 0 \\ -7.26\text{E}-7 & 3.77\text{E}-7 & 0 \\ 0 & 0 & 1.11\text{E}-5 \end{pmatrix} \tag{16}$$

$$\begin{pmatrix} 8.36\text{E}-6 & 7.02\text{E}-6 & 0 \\ 7.02\text{E}-6 & 8.36\text{E}-6 & 0 \\ 0 & 0 & 6.02\text{E}-6 \end{pmatrix} \tag{17}$$

$$\begin{pmatrix} 1.26\text{E}-6 & -4.34\text{E}-7 & 0 \\ -4.34\text{E}-7 & 5.26\text{E}-7 & 0 \\ 0 & 0 & 1.01\text{E}-6 \end{pmatrix} \tag{18}$$

where Supplementary Equations (13)–(18) correspond to Supplementary Fig. 4a, b, c, d, e, and f, respectively.

Supplementary Figure

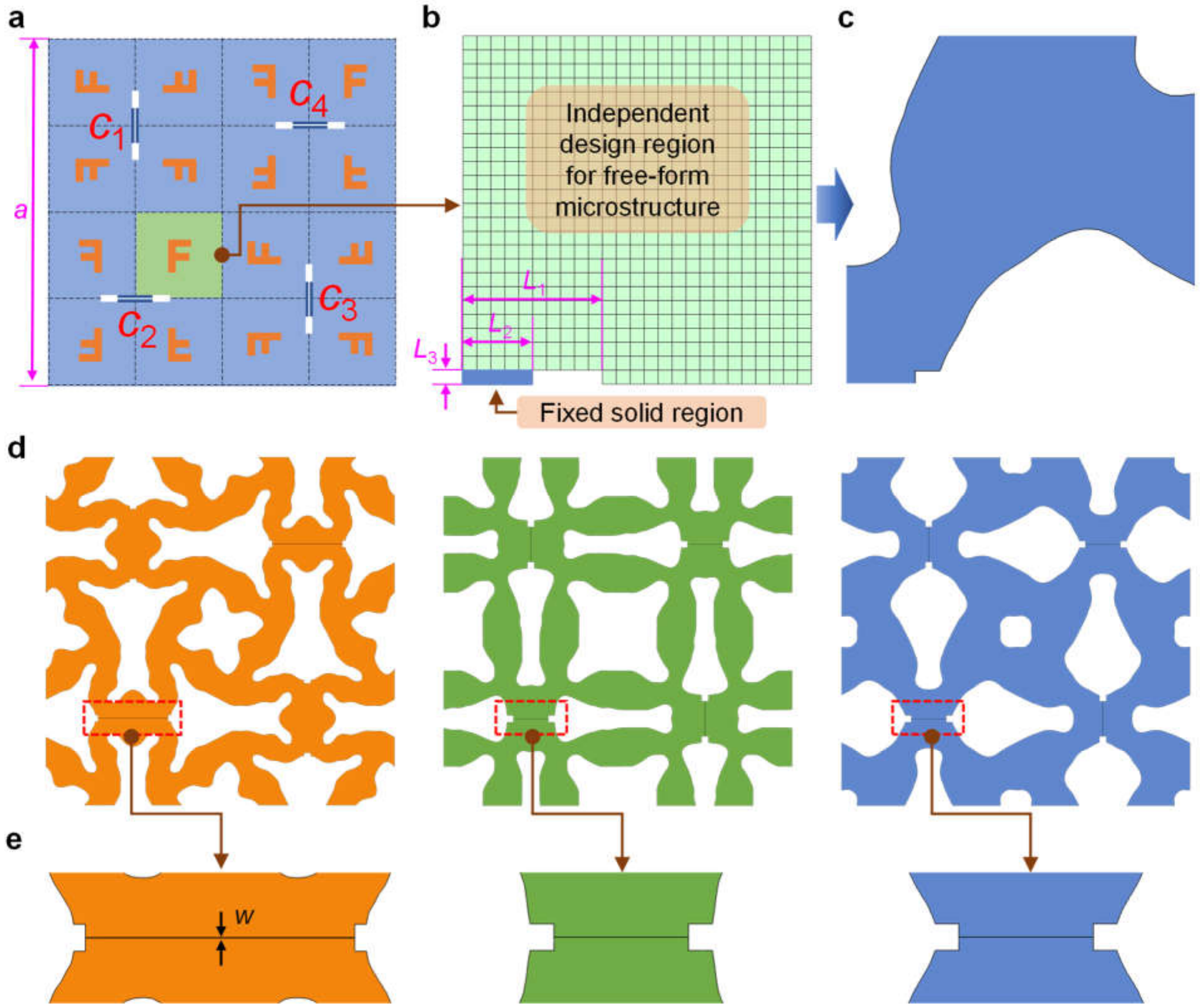

Supplementary Fig. 1: Parameters of predefined slits, designed unit cells, and local view of the slits. **a**, Microstructural symmetry and locations of the predefined slits. The independently designed region is indicated in green, and the orange 'F' is used to indicate the symmetry in the designed region. **b**, Independent design region and fixed solid region for the predefined slits. The unit cell is discretized into about 10000 design elements. The width w of the slits is $0.0002a$. $L_3 = 0.01a - 0.5w$. For the non-reciprocal Poisson's ratio, $L_1 = 0.15a$, $L_2 = 0.1a$. For the ultra-large and step-like Poisson's ratios, $L_1 = 0.1a$, $L_2 = 0.05a$. **c**, Microstructural topology in the independent design region of the metamaterial with step-like Poisson's ratios. **d**, Microstructural topologies of the designed metamaterials with non-reciprocal, ultra-large, and step-like Poisson's ratios, respectively. **e**, Locally enlarged views of the slits in the designed metamaterials with non-reciprocal, ultra-large, and step-like Poisson's ratios, respectively.

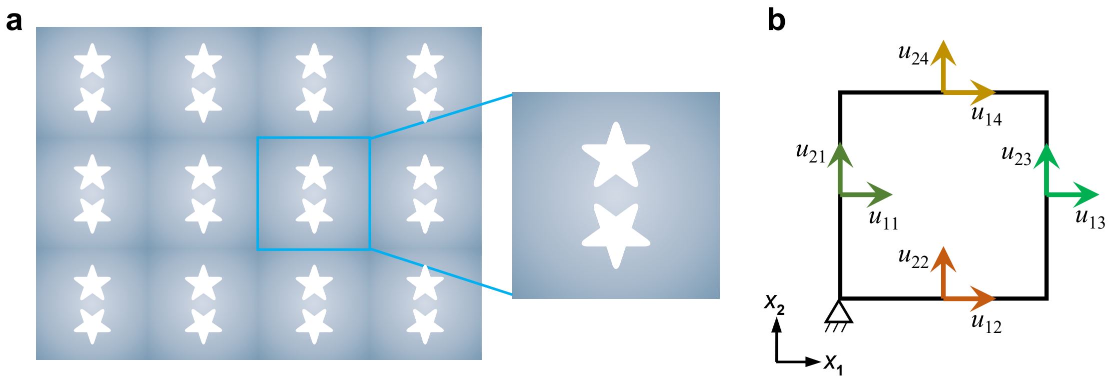

Supplementary Fig. 2: Periodic boundary conditions. **a**, Metamaterial with periodically arranged unit cells. **b**, Periodic boundary conditions: $u_{13} - u_{11} = u_1, u_{21} = u_{23}, u_{24} - u_{22} = u_2, u_{12} = u_{14}$.

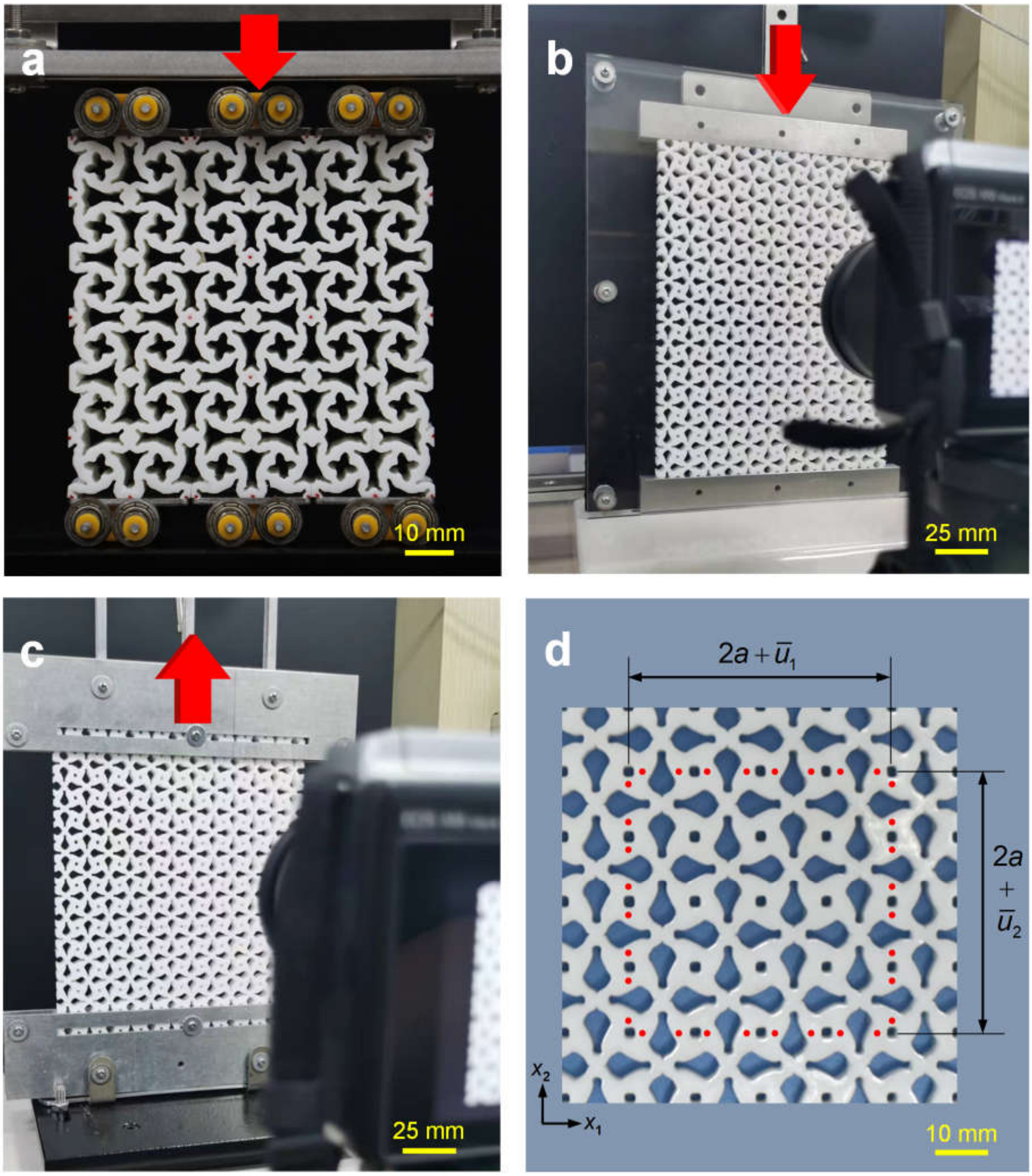

Supplementary Fig. 3: Experiment and measurement. a, Compression test for non-reciprocal Poisson's ratios. **b**, Compression test for step-like Poisson's ratios. **c**,Tension test for step-like Poisson's ratios. **d**, Tracked red points on the sample for calculating Poisson's ratios.

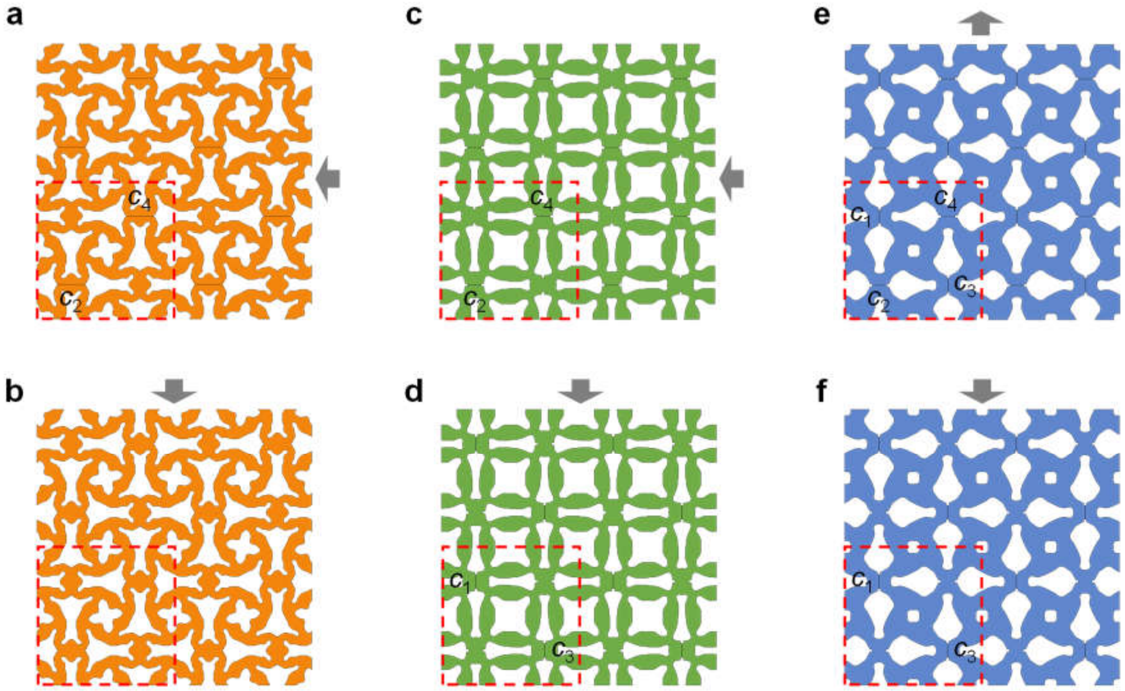

Supplementary Fig. 4: Equivalent models. **a**, Slits c_2 and c_4 are reserved to mimic the metamaterial with non-reciprocal Poisson's ratios under transverse compression. **b**, Slits c_2 and c_4 are replaced by solid connections to mimic a metamaterial with non-reciprocal Poisson's ratios under longitudinal compression. **c**, Slits c_1 and c_3 are replaced by solid connections to mimic a metamaterial with ultra-large Poisson's ratios under transverse compression. **d**, Slits c_2 and c_4 are replaced by solid connections to mimic a metamaterial with ultra-large Poisson's ratios under longitudinal compression. **e**, Four slits are reserved to mimic a metamaterial with step-like Poisson's ratios under tension. **f**, Slits c_2 and c_4 are replaced by solid connections to mimic a metamaterial with step-like Poisson's ratios under compression. Each equivalent model is only valid for a special load case indicated by a gray arrow. All equivalent models were numerically simulated without defining self-contacts.

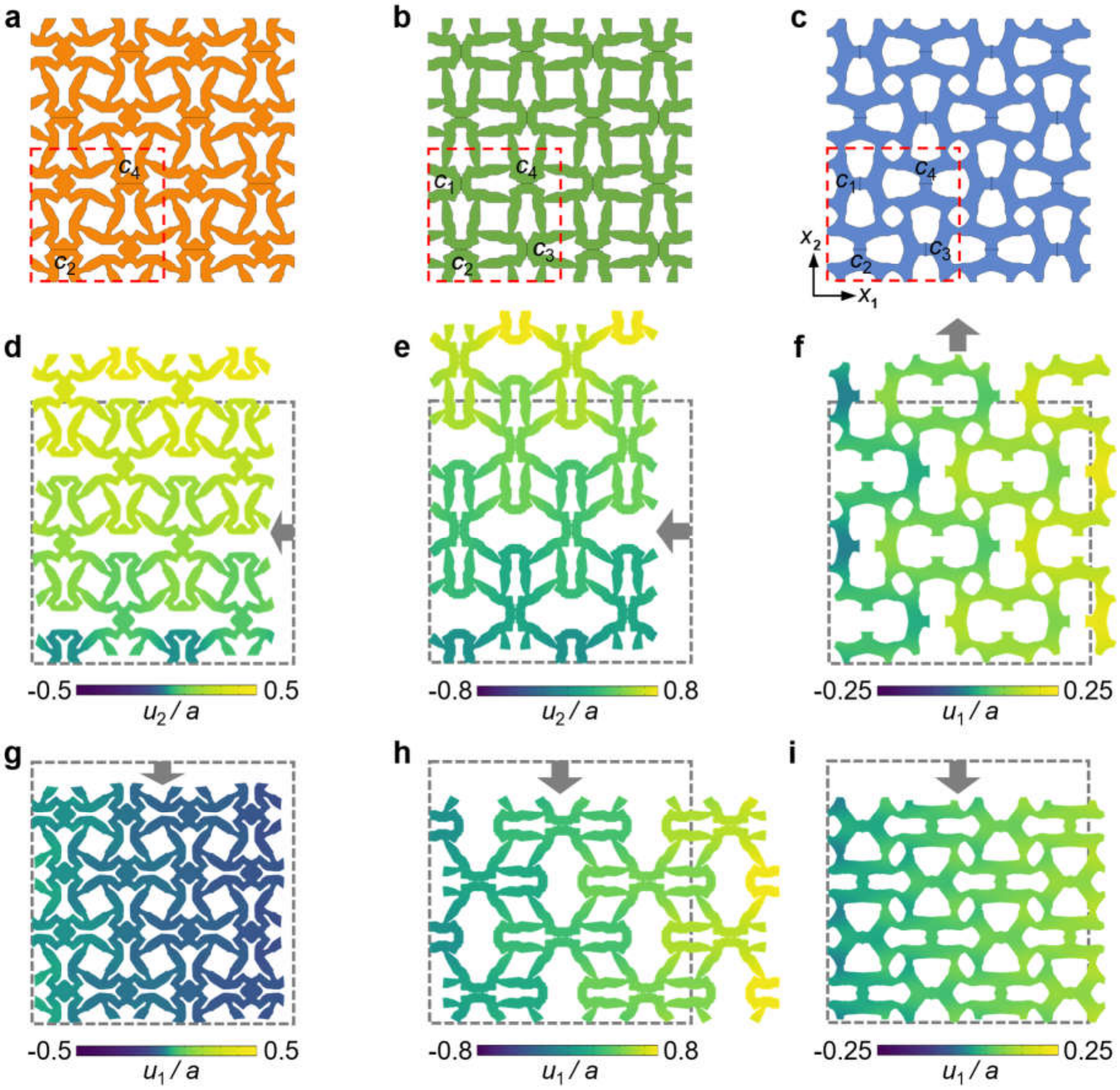

Supplementary Fig. 5: Numerical simulations of metamaterials with other customized Poisson's ratios. a, **b**, **c**, Simulated microstructures with non-reciprocal, ultra-large, and step-like Poisson's ratios, respectively. Each microstructure consists of 2×2 unit cells, one of which is boxed by red dashed lines. Prescribed slits labeled c_1, c_2, c_3, and c_4. **d**, **g**, Deformed microstructures with non-reciprocal Poisson's ratios under transverse and longitudinal compressions (strains of −10%), respectively. **e**, **h**, Deformed microstructures with ultra-large Poisson's ratios under transverse and longitudinal compressions (strains of −15%), respectively. **f**, **i**, Deformed microstructures with step-like Poisson's ratios under longitudinal tension (a strain of 15%) and compression (a strain of −15%), respectively.

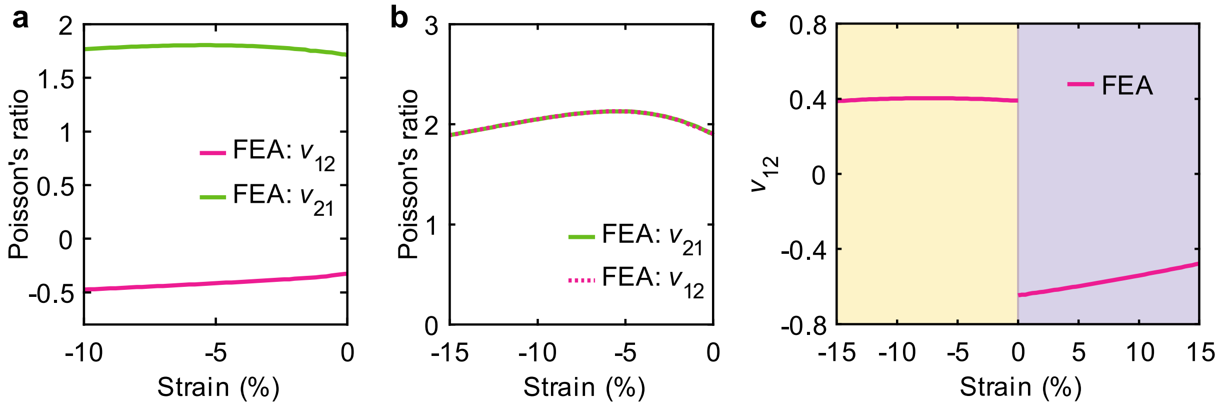

Supplementary Fig. 6: Calculated Poisson's ratios of metamaterials in Supplementary Fig. 5. **a**, Non-reciprocal Poisson's ratios. **b**, Ultra-large Poisson's ratios. **c**, Step-like Poisson's ratios. The two shaded areas correspond to compression and tension strains, respectively.

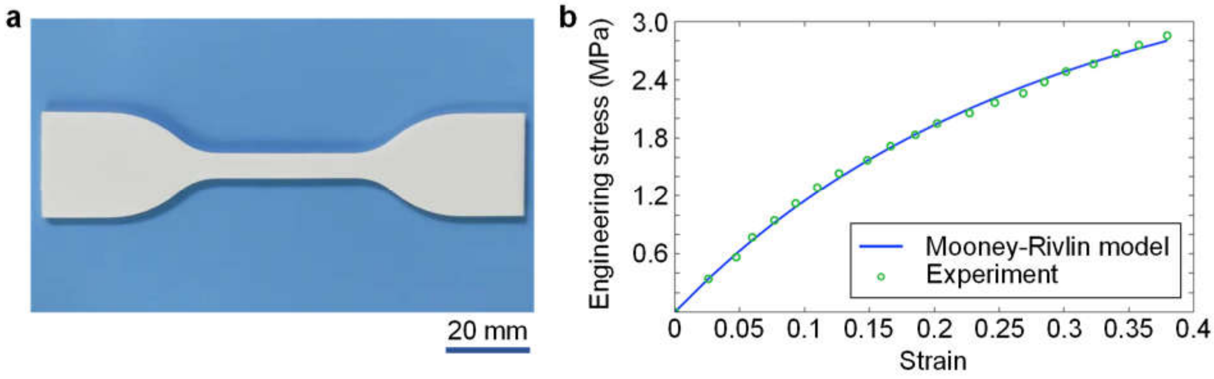

Supplementary Fig. 7: Test of the base material. a, Sample of the base material. **b**, Engineering stress vs. strain from the experiment and the two-term Mooney–Rivlin model.

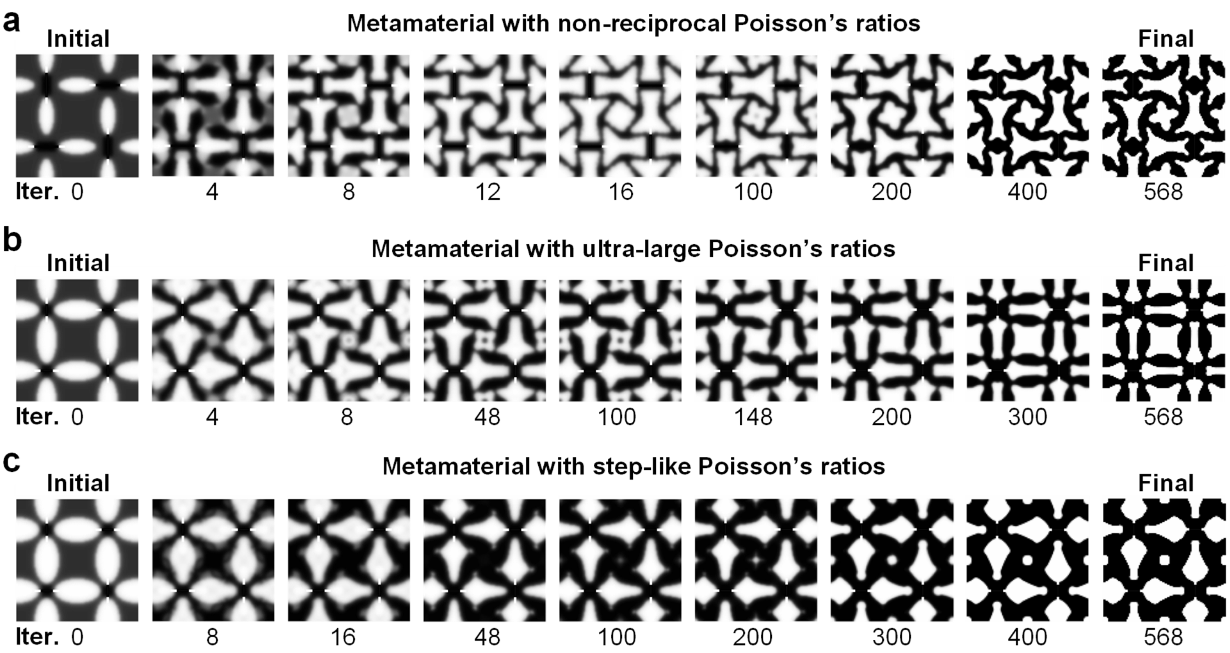

Supplementary Fig. 8: Update processes of microstructural topology for three types of metamaterials. **a**, Non-reciprocal Poisson's ratios. **b**, Ultra-large Poisson's ratios. **c**, Step-like Poisson's ratios. The number of iterations (Iter.) is listed under the microstructures.
