# Supplementary material for: Self-bridging metamaterials surpassing the theoretical limit of Poisson’s ratios
Source: Nat Commun. 2023 Jul 7;14:4041. doi: 10.1038/s41467-023-39792-9 (PMC10328922; doi:10.1038/s41467-023-39792-9)
Supplement: Supplementary file 3 — Description of Additional Supplementary Files [file 41467_2023_39792_MOESM3_ESM.pdf]

Description of Additional Supplementary Files

File Name: Supplementary Movie 1
Description: Experiment of non-reciprocal Poisson's ratios

File Name: Supplementary Movie 2
Description: Experiment of ultra-large Poisson's ratios

File Name: Supplementary Movie 3
Description: Experiment of step-like Poisson's ratios
